# Manual Medium Incision Cataract Surgery with Descemet's Stripping Endothelial Keratoplasty: A Novel Triple Procedure

**DOI:** 10.1155/2015/745409

**Published:** 2015-01-12

**Authors:** Alvin L. Young, Prudence P. C. Chow, Vishal Jhanji

**Affiliations:** ^1^Department of Ophthalmology, Prince of Wales Hospital, The Chinese University of Hong Kong, Shatin, New Territories, Hong Kong; ^2^Alice Ho Miu Ling Nethersole Hospital, Tai Po, New Territories, Hong Kong

## Abstract

*Purpose*. To describe the surgical technique and outcomes of combined Descemet's stripping endothelial keratoplasty and medium incision manual cataract surgery (MICS) in Chinese eyes. *Methods*. Surgery was performed in 8 eyes of 7 patients (5 females, 2 males). Primary outcomes included success of the surgery and final outcomes. *Results*. Surgery was performed in patients with Fuchs' endothelial dystrophy and cataract (mean age 75.5 ± 3.64 years). MICS tunnel was used to insert the donor lenticule into the anterior chamber. All surgeries were performed successfully. Graft dislocation was seen in 1 eye requiring repositioning with intracameral sulfur hexafluoride gas on the first postoperative day. Graft rejection was noted in one patient at the end of one year. The mean decimal best-corrected visual acuity improved from 0.1 ± 0.07 to 0.3 ± 0.15. Suboptimal visual acuity in 2 cases was due to radiotherapy-related optic neuropathy (*n* = 1) and myopic maculopathy (*n* = 1). The mean target spherical refraction was −1.11 ± 0.17 diopters (myopic) and the mean achieved spherical refraction was 1.18 ± 0.87 diopters (hyperopic) resulting in a mean hyperopic shift of 2.2 diopters. *Conclusions*. The approach of combined Descemet's stripping endothelial keratoplasty and MICS is a viable surgical technique for cases with endothelial dysfunction and cataract.

## 1. Introduction

Lamellar corneal transplantation surgery allows for selective replacement of the diseased corneal endothelium [1–4]. Descemet's stripping endothelial keratoplasty (DSEK) and Descemet's membrane endothelial keratoplasty (DMEK) have been shown to have superior outcomes compared to penetrating keratoplasty in terms of postoperative astigmatism and visual function [5, 6]. Fuchs' endothelial dystrophy is one of the main indications for endothelial keratoplasty. However, patients with Fuchs' endothelial dystrophy frequently suffer from coexisting cataract. Cataract surgery has been successfully combined with penetrating keratoplasty since the 1970s and became popular as the “triple procedure.” More recently, the combination of endothelial keratoplasty with cataract surgery was named the “new triple procedure” [7]. Advantages of the triple procedure include faster visual rehabilitation and no further endothelial damage induced by sequential cataract surgery. In the classic triple procedure, penetrating keratoplasty induces changes in the anterior and posterior corneal curvature with reduced predictability because of an alteration in the keratometric readings needed for intraocular lens power calculation. This leads to the increased likelihood of postoperative refractive errors and anisometropia. DSEK on the other hand induces no significant changes in corneal topography and therefore the refractive outcomes after triple DSEK are more predictable, although a mild postoperative hyperopic shift may be observed [8].

In the present study, we describe a novel surgical approach of combined Descemet's stripping endothelial keratoplasty and medium incision cataract surgery (MICS).

## 2. Materials and Methods

### 2.1. Surgical Technique

**Figure 1:**
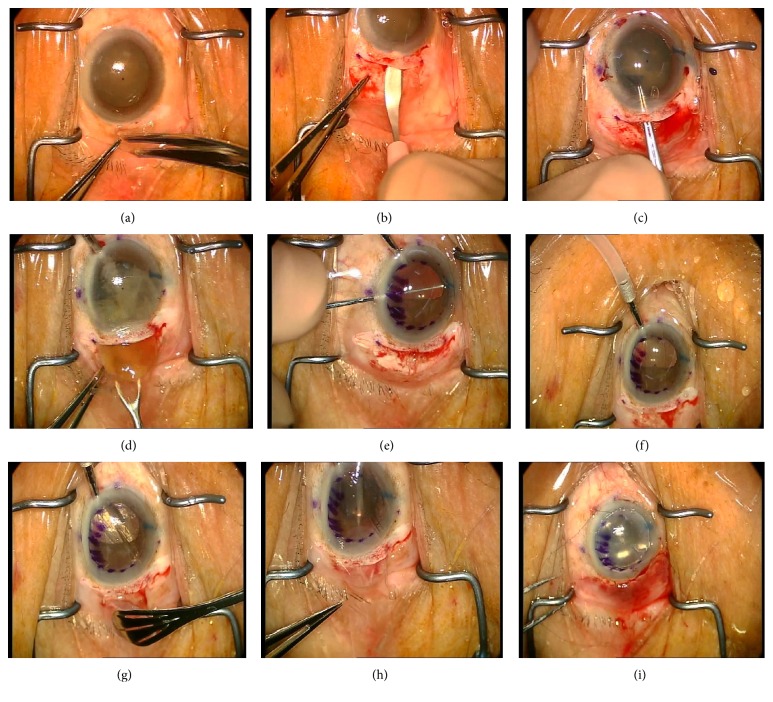
Surgical procedure of combined Descemet's stripping endothelial keratoplasty and medium incision cataract surgery. (a) Conjunctival limbal peritomy from 7- to 11-o'clock positions (for the right eye). (b) An 8 mm long, straight, partial thickness scleral incision was made with a 2.5 mm crescent knife. Using the crescent knife, a 4 mm long sclerocorneal tunnel was dissected from the scleral incision. (c) Anterior capsulorhexis was performed using a pair of capsulorhexis forceps or a bent 27-gauge needle. (d) Nucleus was delivered with a vectus. (e) Descemet's membrane was stripped with a reverse Sinskey hook utilizing the epithelial mark as a guide. (f) A 5 mm Sheet glide was inserted into the anterior chamber through the scleral wound. (g) The donor lenticule was transferred endothelial side down onto the viscoelastic-coated Sheet glide. (h) A pair of forceps was utilized to draw the donor lenticule into the anterior chamber through the nasal paracentesis. (i) At the end of the surgery, the anterior chamber was filled with air for 8–10 minutes.

All surgeries were performed under retrobulbar anesthesia using a 1 : 1 mixture of 2% lignocaine and 0.5% bupivacaine. A temporal approach was used. Conjunctival limbal peritomy was started with two 3 mm oblique relaxation cuts at the 7- and 11-o'clock positions (for the right eye). This was followed by a 10 to 12 mm limbal peritomy (Figure 1(a)). An 8 mm long, straight, partial thickness scleral incision with its center 2 mm from the limbus was made with a 2.5 mm crescent knife (Alcon Surgicals, Fort Worth, TX, USA). Using the crescent knife, a 4 mm long sclerocorneal tunnel was dissected from the scleral incision, extending approximately 2 mm into clear cornea (Figure 1(b)). Three paracentesis sites were created with a 15°  slit knife at the 3-, 6-, and 12-o'clock positions (for the right eye). 0.1 mL of trypan blue (0.6 mg/mL) (VisionBlue; Dutch Ophthalmic Research Center, Amsterdam, Netherlands) was used to stain the anterior capsule under air. A dispersive viscoelastic agent (Viscoat, Alcon Laboratories Inc.) was used to reform the anterior chamber. The anterior corneal surface was marked using an 8 mm marker. A 3 mm keratome (Alcon Surgicals, Fort Worth, TX, USA) was introduced into the center of the tunnel, and the anterior chamber was entered. Anterior capsulorhexis was performed using a pair of capsulorhexis forceps or a bent 27-gauge needle (Figure 1(c)). A gentle hydrodissection was performed to loosen the attachments of the nucleus to the capsular bag. Two Sinskey hooks were used to dislocate the nucleus from the bag as described previously [9].

The internal entry across the entire length of the sclerocorneal tunnel was completed with a keratome. An anterior chamber maintainer was then inserted into the distal paracentesis wound. Nucleus delivery was completed with a vectus (Figure 1(d)). Subsequently, irrigation and aspiration were performed using a 23-gauge aspiration cannula (Duckworth & Kent Ltd., Baldock, Hertfordshire, England). The anterior chamber was filled with a cohesive viscoelastic agent (ProVisc, Alcon Laboratories Inc.). An intraocular lens was inserted in the capsular bag. With the AC maintainer infusion on, the residual viscoelastic agent was removed using the aspiration cannula. Descemet's membrane was stripped with a reverse Sinskey hook utilizing the epithelial mark as a guide (Figure 1(e)). Descemet's membrane was removed in total.

Donor lenticules were prepared either manually in DSEK or by microkeratome in Descemet's stripping automated endothelial keratoplasty (DSAEK). In DSEK, the donor's cornea was mounted in a pressurized artificial anterior chamber filled with Optisol. Manual dissection was initiated in the periphery with a diamond knife, which was calibrated to a desired depth. The corneal stromal dissection was completed with a crescent knife. The donor lenticule was then placed endothelial side up and trephination was performed from the endothelial side. The anterior stromal cap was carefully separated from the underlying donor lenticule. For DSAEK, precut eye bank-prepared tissue was available from the local eye bank.

A 5 mm Sheet glide was inserted into the anterior chamber through the scleral wound (Figure 1(f)). Viscoelastic agent (Healon, Advanced Medical Optics, Santa Ana, CA) was placed on the surface of the glide. The donor lenticule was transferred endothelial side down onto the viscoelastic-coated Sheet glide (Figure 1(g)). In seven out of the eight cases, a pair of Tan's forceps (Asico, Westmont, USA) was utilized to draw the donor lenticule into the anterior chamber through the nasal paracentesis (Figure 1(h)). During this maneuver, anterior chamber was maintained via the infusion line. The infusion line was removed after the graft was pulled into the anterior chamber. In one case, the donor lenticule was inserted after being taco-folded and was then unfolded inside the anterior chamber with the injection of filtered air. At the end of the surgery, the anterior chamber was filled with air for 8–10 minutes (Figure 1(i)). Approximately 50% of the air was left in the anterior chamber. The side ports were hydrated, and the main wound was sutured with interrupted 10-0 nylon sutures when needed.

## 3. Results and Discussion

Combined EK and MICS surgery was performed in 8 eyes of 7 patients (5 females, 2 males) with Fuchs' endothelial dystrophy and cataract. The mean age of the patients was 75.5 ± 3.64 years (Table 1). Endothelial graft was prepared manually in 4 eyes and with microkeratome in 4 eyes. Surgery was performed successfully in all eyes. There were no intraoperative complications. Graft dislocation was seen in 1 eye requiring repositioning with intracameral sulfur hexafluoride (18%) gas on the first postoperative day. One case had mild inferior graft displacement that did not require any surgical intervention. Graft rejection was noted in one patient at the end of one year. Quick resolution of graft rejection was achieved with intensive corticosteroids.

The mean decimal best-corrected visual acuity (Snellen) improved from 0.1 ± 0.07 to 0.3 ± 0.15. Six out of 8 cases (75%) achieved a best-corrected visual acuity of ≥0.3 at the end of 1 year. Suboptimal visual acuity in 2 cases was due to radiotherapy-related optic neuropathy (*n* = 1) and myopic maculopathy (*n* = 1). Two cases had developed posterior capsular opacification postoperatively.

Sanders—Retzlaff—Kraff/Theoretical (SRK/T) formula was employed for the calculation of intraocular lens power before the surgery. The mean target spherical refraction was −1.11 ± 0.17 diopters (myopic) and the mean achieved spherical refraction was 1.18 ± 0.87 diopters (hyperopic) resulting in a mean hyperopic shift of 2.2 diopters.

Selective corneal transplantation has slowly replaced the conventional “one for all” full-thickness keratoplasty [10]. Consequently, the definition of a triple procedure has been expanded to include EK with cataract surgery. However, it may not be feasible to perform phacoemulsification in all cases undergoing a triple procedure due to the lack of resources (especially in developing economies) or expertise (very dense mature cataracts) or both. Both phacoemulsification and small-incision cataract surgery have been shown to be safe and effective for visual rehabilitation of cataract patients [11]. One prospective, comparative study found that although the effectiveness of MICS and phacoemulsification was not significantly different, phacoemulsification was associated with higher costs [12]. Similar observations were made in other studies [13]. It is often difficult to perform phacoemulsification in cases with severe corneal edema, which impairs adequate intraoperative visualization. Our novel approach of combined EK and MICS provides an effective solution to these issues, since the approach and surgical steps are inherently complementary to each other. The MICS technique used in our study has been described earlier [9]. The highlight of this surgical technique includes the use of a large scleral tunnel fashioned temporally in order to provide easy access during the surgery. This is pertinent in Chinese eyes with small palpebral fissures and shallow anterior chamber depth [3]. In our study, cataract surgery was performed through a sutureless scleral tunnel. After cataract extraction and intraocular lens implantation, we used the same scleral tunnel to insert the donor lenticule. The use of a large scleral tunnel and an anterior chamber glide does not require folding of the graft thereby minimizing the risk of endothelial trauma. Successful use of scleral tunnel during EK has been described previously using Busin's glide [14]. However, we feel that it is challenging to introduce Busin's glide into the anterior chamber in Chinese eyes that are characteristically small and have a shallow anterior chamber. Hence, we have adopted the use of pull-through technique and anterior chamber Sheet glide for graft insertion at our center. In the present study, all surgeries were performed successfully using the same surgical technique without any intraoperative problems. A good visual outcome was achieved in majority of the cases.

One case (12.5%) suffered donor dislocation in our case series. This may be related to retained viscoelastic agent in the donor-host interface. Although we tried to minimize the use of viscoelastic agent during the donor insertion, it is not possible to entirely eliminate the use of viscoelastic agents during EK using a Sheet glide. Nevertheless, intracameral sulfur hexafluoride was used to successfully reattach the donor lenticule. Graft dislocations after EK triple procedure have been reported to occur in 1.77% of the cases by Terry et al. [15] and up to 14.3% of the cases by Covert and Koenig [7]. Suh et al. reported the results of EK in 118 eyes of which a combination of phacoemulsification and EK was performed in 41 eyes [16]. Overall, graft detachment was the most commonly observed complication (23% of eyes) in their study [16]. However, the authors did not specify the proportion of graft dislocations after triple procedure. Although it is believed that the risk of graft dislocations is low with the use of anterior chamber maintainer thereby avoiding viscoelastic during EK surgery [14], a large, randomized, prospective study of EK surgery with or without the use of viscoelastic agent can verify the relative rates of tissue dislocation resulting from this single factor [15].

In our study, BCVA of 0.3 or better was achieved in 6 out of 8 cases. Visual outcomes were suboptimal in 2 cases due to associated problems in the posterior segment. Terry et al. reported a best spectacle corrected visual acuity of 0.5 or higher in 97% of the patients at the end of 12 months [15], while another study reported excellent visual outcomes (average 20/34) after EK triple procedure [7]. The visual outcomes after triple procedure have generally been reported to be good. One of the unresolved issues is the preoperative detection of posterior segment problems especially cystoid macular edema that might affect the visual acuity outcomes in these patients. Similar to conventional triple procedure, careful preoperative retinal screening (if the corneal clarity permits an adequate view of the posterior segment) should be performed.

In the present study, the mean target preoperative spherical refraction was 1.1 diopters (myopic). However, a mean hyperopic shift of more than 2 diopters was observed postoperatively. Because early experience with EK had demonstrated a trend towards postoperative hyperopic shift in refraction of approximately 1 diopter, eyes undergoing a triple procedure in our series were actually targeted between −0.89 and −1.34 diopters preoperatively. However, only two out of eight eyes included in this study achieved plano refraction. Our findings match the earlier report by Covert and Koenig [7] who reported a mean hyperopic shift of 1.13 diopters in their cases. Terry et al. however did not observe any significant hyperopic shift in their cases that underwent triple procedure [15]. The hyperopic shift observed in our study may be attributed to the learning curve in terms of intraocular lens power selection during EK triple procedure at our centre. The graft thickness would be variable in our cases that underwent DSEK with manual preparation of the donor lenticule. Another possibility is inaccurate preoperative keratometry values in these cases due to irregular corneal surface.

## 4. Conclusions

Overall, our novel approach of combined EK and MICS can be performed for patients with Fuchs' corneal dystrophy and dense cataract. The limitations of the current study include a small sample size and retrospective design. We did not have preoperative data for all our patients. Further studies with large sample size, a comparative group, and long-term outcomes are desirable.

## Figures and Tables

**Table 1:** Surgical outcomes for patients who underwent combined Descemet's stripping endothelial keratoplasty and medium incision cataract surgery.

Age/sex	Pre-op BCVA	Surgery	Donor graft (diameter; thickness, endothelial cell count/mm^2^)	Recipient pre-op pachymetry (microns)	Post-op complications	Final VA	Target refraction	Final refraction	Pre-op ECD	Post-op ECD (months post-op)	Remarks
75/F	0.1	DSEK + MICS + IOL	7.5 mm; N/A, 2638	574	Partial graft dislocation	0.3	−1.27	Plano	NA	NA	Vascular dementia
69/M	0.1	DSEK + MICS + IOL	8 mm; N/A, 3174		Nil	0.1	−0.93	+1.75	NA	663 (24 m)	NPC with RT 1999; temporal lobe necrosis
75/F	0.1	DSEK + MICS + IOL	8 mm; N/A, 2597	NA	Nil	0.5	−1.01	Plano	NA	1453 (0.5 m)	
76/F	0.2	DSAEK + MICS + IOL	8 mm; 171 µm, 2544	NA	Graft rejection noted after 1 year	0.3	−1.34	+2.00	NA	NA	Graft rejection
75/M	0.2	DSAEK + MICS + IOL	8 mm; 114 µm, 2570	564	Nil	0.4	−1.16	+2.00	NA	524 (6 m)	
81/F	0.2	DSAEK + MICS + IOL	8 mm; 118 µm, 2597	NA	Mild inferior graft displacement	0.5	−1.31	+2.00	644	519 (12 m)	
78/F	0.001	DSAEK + MICS + IOL	7.5 mm; 107 µm, 2666	NA	PCO	0.3	−0.89	+1.00	NA	1535 (3 m)	
72/M	0.1	DSEK + MICS + IOL	8 mm; N/A, 2702		PCO	0.1	−1.03	+0.75	948	1925 (3 m)	NPC with RT 1999; temporal lobe necrosis

M: male.

F: female.

DSEK: Descemet's stripping endothelial keratoplasty.

DSAEK: Descemet's stripping automated endothelial keratoplasty.

MICS: medium incision cataract surgery.

IOL: intraocular lens.

BCVA: best-corrected visual acuity.

PCO: posterior capsular opacification.

NPC: nasopharyngeal carcinoma.

RT: radiotherapy.

SEQ: spherical equivalent.

ECD: endothelial cell density.
